# Population Norms for Hand Grip and Precision Grip Strengths in Polish Children and Adolescents Aged 3–19

**DOI:** 10.3390/jcm13164833

**Published:** 2024-08-16

**Authors:** Michał Górecki, Marta Kazarców, Agnieszka Protasewicz, Piotr Czarnecki, Leszek Romanowski

**Affiliations:** Department of Traumatology, Orthopaedics and Hand Surgery, Poznań University of Medical Sciences, 61-545 Poznań, Polandpiotr_czarnecki@tlen.pl (P.C.); romanowski.kcr@gmail.com (L.R.)

**Keywords:** hand grip strength, pinch strength, children, adolescents, norms

## Abstract

**Background:** Normative data on hand and precision grip strengths are essential for evaluating the level of development, the efficacy of rehabilitation, and treatment results. The need for established norms of grip strength in Polish children is one of the problems that Polish physiotherapists and physicians face when treating upper limbs. The aim was to establish normative values of hand and precision grips strengths in Polish children and adolescents aged 3–19. **Methods:** In the years 2012–2023, a sample of 358 children and adolescents with no history of upper limb injuries or congenital upper extremity defects were randomly chosen from kindergartens, primary schools, middle schools, and high schools. They were living in urban agglomerations and in smaller towns or villages. Hand and precision grips like the pincer, three-point, side, and opposition grip strength were assessed using a hand dynamometer and pinchmeter in standard positions. **Results:** The strength in all types of examined hand grips increases with chronological age in both genders. The grip strength was higher in the boys’ group than in the girls’ and it was higher in the right hand than in the left, but the difference was no more greater than 10%. Detailed data with standard deviation were presented in the form of a table, divided by age and sex. **Conclusions:** Norms for grip strength were provided for Polish children and adolescents aged 3–19, allowing therapists and physicians to compare Polish patients with that of normally developed, healthy children of the same age and sex.

## 1. Introduction

Proper grip strength is essential for the functionality of the hands, playing a critical role in managing a broad spectrum of daily activities from early childhood. These activities encompass fundamental tasks such as self-care, eating, schoolwork, and sports. Assessing grip strength in children and adolescents is crucial for determining the level of hand development or the presence of disabilities, particularly in cases of congenital defects [1,2]. In clinical practice, these measurements are often employed in adults to evaluate hand strength following various injuries or conditions, including hand trauma, nerve compression injuries or neuropathies, tendon injuries, wrist instability, and a range of overuse or degenerative diseases [3,4,5,6,7,8,9]. The assessment of grip strength is typically performed prior to treatment to establish a baseline that can be used to gauge the efficacy of surgical interventions and rehabilitation strategies for the upper limb [10,11]. Despite its importance, there is a paucity of studies presenting normative data for grip strength in children across different regions of the world. Those that do exist highlight significant differences in grip strength norms across countries, cultures, and socioeconomic backgrounds. This variation underscores the necessity for healthcare professionals to establish region-specific norms for grip strength [12,13,14,15,16,17,18,19,20,21].

Additionally, the ongoing secular trend in accelerated growth among children further emphasizes the need to periodically update these norms [22].

Recent research has demonstrated that genetic factors significantly influence hand grip strength. Numerous studies have pinpointed specific genetic variants associated with muscle strength. For instance, Willems et al. analyzed over 140,000 participants from the UK Biobank, identifying 16 common genetic variants correlated with grip strength. These variants are situated within or adjacent to genes essential for muscle fiber structure and function, as well as for neuromuscular communication. This evidence indicates that genetic variation can modulate muscle strength and highlights potential avenues for therapeutic interventions targeting muscle weakness [23]. Additionally, Chan et al. underscore the heritability of grip strength, with estimates suggesting that genetic factors account for approximately 30% to 65% of the variability in grip strength. This genetic influence is notably more significant in mid- to late life, where specific genetic markers have been found to affect physical functioning and the trajectory of grip strength decline [24].

Research indicates that cultural influences have a substantial impact on hand grip strength. Variations in grip strength across different populations can be attributed to diverse cultural and lifestyle factors.

Dodds et al. (2016) conducted a systematic review and meta-analysis revealing that grip strength tends to be lower in developing regions, such as parts of Africa, Asia, and Latin America, compared to developed areas like North America, Europe, and Japan. These differences are often linked to variations in nutrition, physical activity, and overall health status between regions. This study suggests the need for region-specific normative data to accurately assess grip strength and related health outcomes [25].

Cultural practices related to occupation and recreational activities also significantly affect grip strength. For example, individuals involved in manual labor or sports that heavily use their hands typically demonstrate higher grip strength than those in less physically demanding roles. This highlights the importance of considering occupational and recreational factors when evaluating grip strength [26]. Additionally, within-country variations in grip strength have been observed, influenced by lifestyle factors such as diet and exercise habits. For instance, older adults in Singapore were found to have weaker grip strength compared to their Western and other Asian counterparts, potentially due to differences in dietary practices, physical activity levels, and healthcare access [25].

These findings underscore the necessity for culturally tailored health assessments and interventions that consider the specific lifestyle and occupational practices of different populations to accurately evaluate and improve grip strength and overall health [25,26].

To date, few studies have reported on reference values for grip strength in the Polish population. The existing studies are limited in scope: one focuses on older adults over the age of 65, another examines children within a narrow age range of 9–11 years, and a third assesses young adult women [27,28,29]. Moreover, the manufacturer of dynamometers and pinchmeters by Biometrics Ltd. E-LINK, Newport, UK, whose products are available in the Polish market, has not provided comprehensive normative data for grip strength in Polish children and adolescents. This gap in the literature and available resources necessitates the establishment of updated, region-specific norms.

The primary objective of this study is to establish reference data for grip strength among Polish children and adolescents aged 3–19 years using the abovementioned equipment. This study aims to fill the existing gaps by providing comprehensive and precise normative data, which can be utilized by physicians and therapists to better assess and treat hand functionality in this population.

Our research will not only contribute valuable data for clinical assessments but will also enhance the understanding of grip strength development in Polish children and adolescents. By using standardized equipment and methodologies, we aim to ensure the reliability and validity of our findings, thereby facilitating their application in both clinical and research settings.

In summary, this study addresses a critical need for updated and region-specific normative values on grip strength in Polish children and adolescents. The establishment of these norms will aid healthcare providers in accurately assessing hand functionality and development, ultimately improving the diagnosis, treatment, and rehabilitation of upper limb conditions in this population. However, it should be remembered that setting population standards is extremely important, not only in groups of children and adolescents, but also in adults.

## 2. Materials and Methods

The study conducted on children and adolescents adhered strictly to ethical guidelines and the principles outlined in the Declaration of Helsinki. Before the commencement of the study, the entire process was thoroughly explained and presented to both the parents and the children. Participation in the study was entirely voluntary, and verbal consent was obtained from all participants and their legal guardians, which was most often one of the parents. Given that the study did not involve any medical experiment, the Local Bioethics Committee issued an opinion that the committee’s consent was not necessary for this type of study. The focus of the study was solely on measuring grip strength using non-invasive methods, ensuring the safety and comfort of all participants throughout the process.

Participants in this study included 358 children and adolescents (181 males and 177 females) from randomly chosen kindergartens, primary schools, middle schools, and high schools, attended by children of different socioeconomic and cultural status, mostly living in the center of the urban agglomeration and also on its outskirts in smaller towns or villages. Only healthy children who expressed a desire and commitment to participate in the examination, and who had no history of upper limb injuries or congenital upper extremity defects that might have influenced hand strength at the time of testing, were included in the study. This careful selection process ensured that the data collected were representative of normal, healthy hand development. The study protocol did not consider the dominant hand or body weight of the study participants.

All measurements were collected from the years 2012 to 2023 and obtained in the Functional Testing Laboratory of the Hand Surgery Department during daytime hours from 10 am to 3 pm. This timing was chosen to ensure consistency in the testing environment and to minimize the impact of daily fatigue or other external factors on hand grip strength. The assessments utilized advanced tools provided by Biometrics Ltd. E-LINK, Newport, UK; we used a Hand Accessory Kit (H400s) (Nine Mile Point Ind. Estate, Cwmfelinfach, Gwent, UK), specifically comprising the Dynamometer (G100) and Pinchmeter (P100).

Grip strength was measured following the standard testing position recommended by the American Society of Hand Therapists (ASHT). This standardized position is widely used in numerous studies to ensure reliable and comparable results [16,30,31,32]. The position required participants to sit with their feet flat on the floor, their shoulder adducted and neutrally rotated, the elbow at 90 degrees of flexion, the forearm in a neutral position, and the wrist extended between 0 and 30 degrees, with an ulnar deviation between 0 and 15 degrees. Importantly, the hand and forearm were not supported during testing. The dynamometer was held upright in line with the forearm to accurately measure hand grip strength.

The Dynamometer G100 offers five standard test positions, from position 1 (smallest spacing) to position 5 (largest spacing). The children performed three trials for each hand, and the arithmetic mean of these trials was used as the maximal score for the global grip strength [33,34]. Children aged 3–6 years were tested in position 1, those aged 7–13 years in positions 1, 2, and 3, and individuals aged 14–19 years in positions 1, 2, 3, 4, and 5. This stratification ensured that the grip strength measurements were appropriately challenging and accurate across different developmental stages.

In addition to global hand grip strength, the study also assessed precise hand grips, including the pincer grip, the three-point grip, and the side grip (key grip). These precise grips were tested on all study participants, and the scores for each precise grip were calculated as the arithmetic mean of three trials. For the thumb opposition grip, one trial was performed for each combination: thumb—index finger, thumb—middle finger, thumb—ring finger, and thumb—little finger. All results were meticulously recorded in kilograms.

Considering that the impact of intervals between trials on hand strength measurements has yet to be definitively proven, this study set the interval at 10 s to ensure consistency [30]. This time was determined by physiotherapists who regularly perform this type of measurement at the authors’ workplace to standardize the study protocol. After the grip strength test, the key grip was tested, followed by the three-point grip, the pincer grip, and finally, the thumb opposition grip.

## 3. Results

The norms for the strength of each hand grip type are categorized by age, sex, and tested hand. These categories include key grip, three-point grip, pincer grip, thumb opposition for individual fingers, and global hand grip (positions 1 to 5). The data provide arithmetic means with standard deviations for age groups 3–19 years (key, three-point, pincer, thumb opposition for individual fingers, global hand grip-position 1), 7–19 years (global hand grip-positions 2 and 3), and 14–19 years (global hand grip-positions 4 and 5).

The grip strength exhibited a broad range, from 3.59 kg in the left hand of three-year-old females to 47.37 kg in the right hand of a 19-year-old male. A significant and gradual increase in grip strength was observed with advancing age. The highest grip strength scores were recorded in testing positions two and three for subjects aged 7–19.

The results are presented in Table 1.

For precise hand grips (key, three-point, pincer grips), the results were consistent across the age groups, ranging from 0.88 kg in the left hand of three-year-old females to 8.75 kg in the right hand of 18-year-old males. The results are presented in Table 2.

Thumb opposition grip strength was the highest between the thumb and index finger, ranging from 0.94 kg in the left hand of three-year-old females to 7.10 kg in the right hand of 18-year-old males, and it was the lowest between the thumb and little finger, ranging from 0.36 kg in the left hand of three-year-old females to 2.40 kg in the right hand of 19-year-old males. The results are presented in Table 3.

The increase in both the global and precise grip strength correlated positively with advancing age in both the right and left hands (Spearman’s rank correlation coefficient with *p* < 0.05). Boys consistently demonstrated a stronger grip strength than girls across all types of hand grip, and the right hand was stronger than the left hand (Mann–Whitney test *p* < 0.05). However, the difference in grip strength between the right and left hands was less than 10%, favoring the right hand.

These findings underscore the importance of age-specific and sex-specific norms in assessing hand grip strength. The data highlight that boys generally possess greater grip strength compared to girls, and there is a natural dominance of the right hand over the left hand.

## 4. Discussion

This study presents normative data for global and precise grip strengths in children and adolescents aged 3–19 years in correlation with sex and age. Our data confirm progressive increases in all types of hand grip in both sexes with advancing age. These results also correspond with studies previously published in other countries [14,16,19,21]. At around 11–12 years of age for females and 12–13 years for males, a significant increase in strength occurred, probably related to the increase in strength that occurs with the onset of maturity [35].

Due to the limited amount of data in this area, we could only partially compare our results with those described in the literature regarding the Polish population of children and adolescents.

The first study assessed hand grip strength, then compared this to the blood lead levels in school-aged children between 9 and 11 years for boys and 9 and 10 years for girls. The average result of the grip strength in the assessed age ranges was 15.40 kg for boys and 10.88 kg for girls; this result is lower by 7% for boys and as much as 20% for girls compared to the average strength described in our study in the same age range. The position during the hand grip was the same as during our measurements. However, the authors of the compared study only performed two grip strength measurements and selected the better result for analysis. It might seem that with such an assumption, the results should be better than when calculating the arithmetic mean from three measurements, as in our study. The lower grip strength could result from the nature of the study, where the authors ultimately assessed the impact of blood lead levels on global grip strength, finding that increased concentration causes a weakening of strength. The studied children, due to residing in areas of Poland prone to pollution, were initially burdened with a risk factor compared to our study group [28].

The second Polish study assessed the grip strength in female students, whose average age was 22 years +/− 1 year [29]. The average grip strength in the studied group was 29.70 kg. Unfortunately, the oldest age group in our study was 19 years, where the average global grip strength in women was just under 26 kg, which is 12.5% lower than in the compared group. Although the patients were nearly four years older, this age difference should not necessarily significantly impact the considerable differences in grip strength [10]. The position during the hand grip was the same as during our measurements. However, the authors of the compared study only performed two grip strength measurements. They selected the better result for analysis, which might have significantly influenced the better results in the compared group of patients.

Studies from the literature were assessed to compare the results obtained in our study against those from countries in different parts of the world and with different cultures: Scandinavia, Western Europe, North America, and the Middle East. The first concerned the grip strength of children in Sweden aged 4–16 years [19], the second involved children in the Netherlands aged 4–14 years [36], the third concerned children from the United States of America aged 6–19 years [16], and the last one assessed grip strength in children from Saudi Arabia aged 6–12 years [37].

In all compared studies, the position during the hand grip test was the same as during our measurements based on the recommendation of the American Society of Hand Therapists. The authors performed three trials for each subject, calculating the average as the result; just as in our study, but in the Netherlands paper, the authors performed only two attempts. Also, the time between the trials was different from 10 s [19,36], as in our protocol, to 1 min [37]. In all the works assessed, the study participants were residents mostly living in the center of the urban agglomeration.

In all age groups, boys were stronger than girls, the right hand was stronger than the left [16,19], and the dominant hand (which was over 90% the right hand) was stronger than the non-dominant [36,37], consistent with our findings. In all age groups, children from Poland had lower grip strength compared to children from the USA and Netherlands but were stronger than those compared to the Swedish and Saudi Arabian study groups.

The largest difference in grip strength was observed in boys aged 6–11 years and girls aged 6–12 years in the USA, which was higher on average by 30% and 28%, respectively, while in the Dutch study, in boys and girls aged 5–9 years, it was higher on average by 16% and 25%, respectively. The difference in grip strength in the American population has decreased significantly to an average of 7–8% in boys after the age of 12 and girls after the age of 13, and in the Dutch population after the age of 9 to an average of 4% in boys and 14% in girls.

On the other hand, children from our study group (both girls and boys) were on average 4–8% stronger than children from Sweden and Saudi Arabia in the age groups studied, where the largest differences, depending on sex, were 21–28% at 6–7 years of age and 17–19% at 10 years of age for Swedish and Saudi children, respectively. Within the Swedish group, this difference decreased after 13 years of age, while in Saudi Arabia it remained at a similar level.

The significant decrease in the difference in grip strength in the assessed groups of children may be due to a developmental leap, which may increase strength with maturity [35]. For children from the USA and Sweden, this was after the age of 12, and for Dutch children, this was after the age of 9. The lack of significant change in the difference in grip strength between children from Poland and Saudi Arabia may be because Omar et al. [37] did not examine children after the age of 12; so, looking at the previously assessed groups, these children could have still not made a developmental leap and achieved a significant increase in strength.

It can be assumed that, based on the assessed works, the highest grip strength was observed in children from the USA, then in the Netherlands, Poland, and Sweden, and the lowest was in Saudi Arabia. This phenomenon, that presents a significant difference in grip strength between children of the same age but raised and maturing in different parts of the world, confirms the fact that strength standards should be established for the population in a given country or even its region due to the significantly important phenomenon of cultural differences as well as the quality of nutrition, physical activity, and overall health status between regions [25].

This study has some limitations. One of them may be the number of 358 subjects, which may seem too small to establish norms. However, according to a paper by Innes et al., there is no indication of an acceptable sample size [30]. Another limitation is that the subjects were mainly from the urban areas and only a few were from suburbs, small towns, or villages, which does not represent a wide range of socioeconomic aspects and cultural differences in Polish individuals. Thus, further study is required using the same standardized procedures in another location within the country. According to some authors, body weight, hand size, and handedness impact hand grip strength, which we did not consider in our study [12,13,14,15,16,17,18,19,20,21]. The above factors may contribute to developing more representative norms for hand grip strength and should be considered in further studies assessing grip strength standards in both children and adults.

However, it is important to remember that establishing population standards is crucial not only for children and adolescents but also for adults, so we therefore recommend further research in this area.

## 5. Conclusions

This study presents age- and sex-specific reference values for hand grip and precision grips strength in Polish children and adolescents. These data will enable physiotherapists and physicians to compare the grip strength of Polish patients with that of normally developed, healthy children of the same age and sex. It can lead to better diagnosis, treatment planning, and rehabilitation outcomes for various conditions affecting the upper limbs.

This work is groundbreaking in that it is the first in the literature to establish normative data for grip strength among children in the Polish population. It serves as a vital supplement to the existing gap in population studies on grip strength in Polish children. Regularly conducting and analyzing such population studies is crucial due to the accelerated growth patterns in children and the socioeconomic changes that have occurred over the decades.

## Figures and Tables

**Table 1:** Average hand grip strength (in kilograms) with standard deviation by sex and tested hand for Polish children and adolescents aged 3–19.

Age	Sex	Global Hand Grip Strength of the Left Hand Position 1	SD	Global Hand Grip Strength of the Right Hand Position 1	SD	Global Hand Grip Strength of the Left Hand Position 2	SD	Global Hand Grip Strength of the Right Hand Position 2	SD	Global Hand Grip Strength of the Left Hand Position 3	SD	Global Hand Grip Strength of the Right Hand Position 3	SD	Global Hand Grip Strength of the Left Hand Position 4	SD	Global Hand Grip Strength of the Right Hand Position 4	SD	Global Hand Grip Strength of the Left Hand Position 5	SD	Global Hand Grip Strength of the Right Hand Position 5	SD
3	F	3.59	1.52	4.08	1.44																
	M	4.52	1.97	4.99	2.20																
4	F	4.29	0.71	4.80	0.87																
	M	4.76	1.17	5.80	1.25																
5	F	5.37	0.88	5.80	1.22																
	M	6.39	1.08	6.58	1.93																
6	F	6.82	1.12	7.12	1.38																
	M	7.95	1.17	8.25	1.31																
7	F	8.75	1.80	8.92	1.66	9.67	1.49	10.16	1.48	8.76	1.14	9.06	1.18								
	M	9.68	1.87	9.97	1.91	10.82	2.02	11.23	1.96	9.82	1.82	10.28	1.99								
8	F	8.53	0.58	8.55	0.78	9.82	1.13	10.04	0.94	9.25	0.92	9.10	0.61								
	M	10.55	2.13	10.91	2.12	12.65	2.48	13.15	2.45	11.49	2.26	11.95	2.43								
9	F	10.39	1.98	11.44	2.12	12.10	2.23	13.71	1.82	11.72	2.16	12.24	1.77								
	M	12.23	2.67	12.96	3.03	14.08	2.30	14.90	2.73	13.55	2.28	14.05	2.73								
10	F	13.23	3.32	13.61	3.27	15.84	3.35	16.75	3.49	15.09	3.47	16.27	3.53								
	M	15.11	1.63	15.31	2.27	17.83	2.25	18.59	2.72	17.20	2.50	17.70	2.70								
11	F	14.89	2.30	14.82	2.79	17.12	2.31	17.30	2.91	16.43	2.60	17.12	2.97								
	M	16.56	1.31	17.02	1.82	19.55	1.90	20.81	2.19	19.23	2.06	20.18	2.45								
12	F	17.13	2.91	17.74	3.13	20.00	3.03	20.25	3.18	19.60	2.83	19.86	3.06								
	M	20.35	3.59	21.61	3.58	22.74	2.81	24.94	3.44	21.45	3.24	22.56	3.04								
13	F	17.65	1.22	18.64	1.45	21.65	1.43	22.10	1.44	20.72	1.19	21.35	1.34								
	M	24.06	2.63	24.34	2.80	28.58	3.50	28.65	3.88	26.18	3.95	27.44	3.87								
14	F	18.87	2.32	19.61	2.64	23.15	2.52	23.64	3.32	22.71	2.64	23.19	2.93	19.93	2.18	19.87	3.01	17.45	1.94	17.45	2.30
	M	24.95	4.03	24.82	4.33	29.20	4.61	30.10	4.62	28.98	4.48	29.55	3.97	25.32	4.69	25.45	4.03	22.58	4.38	22.65	4.00
15	F	20.13	1.40	21.01	1.86	24.16	1.55	25.38	1.55	23.65	2.02	24.71	1.66	20.45	1.44	20.98	1.84	18.41	0.81	18.53	1.31
	M	25.37	3.66	25.36	3.15	30.35	3.92	32.29	3.72	30.31	3.94	32.46	3.93	25.37	3.22	26.02	3.06	22.93	3.14	23.07	2.95
16	F	20.88	2.06	21.84	2.34	26.72	2.21	27.15	2.81	25.88	2.38	26.42	2.75	20.66	1.56	20.98	1.35	19.26	1.54	19.95	2.00
	M	31.36	6.11	30.42	6.17	38.45	5.72	39.74	6.60	37.14	5.18	39.41	6.76	31.43	5.21	31.62	5.84	27.77	4.89	28.64	4.80
17	F	23.67	3.27	23.70	2.08	28.54	2.31	30.39	2.30	28.05	2.22	29.95	2.31	23.94	2.30	23.96	1.15	21.45	2.43	22.12	2.96
	M	32.12	7.13	33.40	7.03	40.57	8.31	41.45	8.93	39.54	7.75	40.69	7.58	32.31	7.09	32.77	7.20	29.19	6.52	30.00	6.67
18	F	23.98	4.39	25.33	3.62	28.26	4.27	28.94	5.00	27.62	4.16	28.23	4.83	24.10	3.89	24.93	4.19	22.94	3.70	23.65	3.35
	M	35.48	8.63	38.86	9.21	41.16	8.09	43.60	8.73	41.32	7.21	42.02	8.53	36.25	7.12	36.64	8.03	33.09	7.08	34.56	7.67
19	F	23.99	3.08	24.24	2.53	30.00	3.42	30.96	3.19	29.32	3.19	29.50	3.47	24.48	3.00	24.23	2.23	21.58	2.52	21.57	2.45
	M	36.62	7.07	37.80	7.30	9.67	1.49	10.16	1.48	8.76	1.14	9.06	8.86	37.45	7.70	38.51	7.94	34.12	7.08	34.46	7.23

F—female, M—male, SD—standard deviation.

**Table 2:** Average key, three-point, and pincer grip strength (in kilograms) with standard deviation by sex and tested hand for Polish children and adolescents aged 3–19.

Age	Sex	Key Grip Strength Left Hand	SD	Key Grip Strength Right Hand	SD	Three-Point Grip Strength Left Hand	SD	Three-Point Grip Strength Right Hand	SD	Pincer Grip Strength Left Hand	SD	Pincer Grip Strength Right Hand	SD
3	F	1.09	0.30	1.25	0.32	1.36	0.32	1.55	0.27	0.88	0.38	1.24	0.30
	M	1.18	0.24	1.45	0.18	1.40	0.41	1.65	0.51	1.04	0.34	1.20	0.40
4	F	1.16	0.25	1.32	0.38	1.63	0.27	1.81	0.38	1.11	0.25	1.30	0.31
	M	1.14	0.19	1.31	0.33	1.77	0.21	1.96	0.28	1.17	0.21	1.37	0.25
5	F	1.09	0.17	1.09	0.20	1.53	0.23	1.66	0.29	0.94	0.15	1.01	0.20
	M	1.39	0.30	1.38	0.39	2.02	0.38	1.95	0.47	1.20	0.25	1.18	0.25
6	F	1.39	0.37	1.54	0.35	2.02	0.55	2.19	0.54	1.13	0.20	1.24	0.10
	M	1.41	0.22	1.58	0.19	2.21	0.56	2.50	0.45	1.30	0.21	1.40	0.24
7	F	2.38	0.51	2.54	0.46	2.64	0.59	2.78	0.59	2.20	0.44	2.25	0.49
	M	2.35	0.48	2.55	0.58	3.11	0.62	3.16	0.64	2.36	0.34	2.46	0.35
8	F	2.71	0.69	2.94	0.86	3.11	0.66	3.24	0.76	2.60	0.47	2.73	0.45
	M	2.74	0.67	2.85	0.71	3.16	0.55	3.24	0.60	2.36	0.65	2.39	0.60
9	F	2.90	0.64	3.05	0.74	3.29	0.54	3.34	0.50	2.55	0.33	2.53	0.36
	M	3.16	0.82	3.48	0.76	3.65	0.66	3.85	0.57	2.58	0.47	2.70	0.46
10	F	3.66	1.24	4.15	1.38	3.88	1.06	4.25	1.21	2.74	0.80	2.97	0.77
	M	3.74	0.72	3.86	0.81	3.85	0.83	4.01	0.77	2.83	0.60	2.95	0.54
11	F	4.23	1.37	4.29	1.44	4.15	0.83	4.30	0.84	3.18	0.71	3.33	0.60
	M	4.23	0.78	4.57	0.88	4.55	0.84	4.66	0.78	3.09	0.48	3.20	0.42
12	F	3.89	0.94	3.97	1.01	4.79	0.79	4.91	0.81	3.52	0.66	3.62	0.72
	M	5.30	0.64	5.51	0.65	6.03	0.61	6.26	0.58	4.25	0.59	4.41	0.68
13	F	4.04	1.00	4.15	1.09	4.85	0.85	5.44	1.09	4.07	0.77	4.18	0.73
	M	5.73	1.35	5.94	1.41	5.92	1.29	6.60	1.24	4.61	1.08	5.03	1.03
14	F	4.60	0.61	4.74	0.77	5.35	1.16	5.82	0.82	4.07	1.03	4.70	1.01
	M	5.09	0.94	5.26	1.03	5.90	1.16	6.26	0.97	4.77	0.83	5.09	0.83
15	F	4.70	0.59	4.85	0.62	5.82	0.73	5.89	0.88	4.25	0.44	4.69	0.62
	M	5.44	0.79	5.72	1.08	6.50	0.53	6.85	0.76	5.10	0.92	5.28	1.18
16	F	5.44	1.51	5.35	1.14	6.43	1.21	6.32	1.12	4.45	1.08	4.79	1.17
	M	6.30	1.79	6.36	1.39	7.40	1.26	7.74	0.86	5.65	1.21	5.44	0.94
17	F	5.95	0.83	6.05	0.83	6.53	1.02	6.58	1.06	4.82	0.93	4.90	0.92
	M	6.75	2.18	6.82	2.19	7.94	2.08	8.29	2.39	6.08	2.00	5.91	1.84
18	F	6.47	0.62	6.35	0.68	6.70	0.52	6.73	0.39	5.03	0.69	5.17	0.64
	M	7.37	1.43	7.40	1.94	8.58	1.99	8.75	2.46	6.55	1.79	5.87	2.64
19	F	6.62	1.02	6.55	0.91	6.77	0.97	6.97	1.11	5.25	0.83	5.47	0.96
	M	7.30	1.48	7.61	1.66	8.54	1.33	8.73	2.00	6.86	0.86	6.86	1.09

F—female, M—male, SD—standard deviation.

**Table 3:** Average thumb opposition grip strength (in kilograms) for individual fingers with standard deviation by sex and tested hand for Polish children and adolescents aged 3–19.

Age	Sex	Thumb Opposition Index Finger Left Hand	SD	Thumb Opposition Index Finger Righ Hand	SD	Thumb Opposition Middle Finger Left Hand	SD	Thumb Opposition Middle Finger Right Hand	SD	Thumb Opposition Ring Finger Left Hand	SD	Thumb Opposition Ring Finger Right Hand	SD	Thumb Opposition Little Finger Left Hand	SD	Thumb Opposition Little Finger Right Hand	SD
3	F	0.94	0.25	1.30	0.37	0.64	0.38	1.01	0.47	0.54	0.26	0.69	0.30	0.36	0.19	0.50	0.26
	M	1.01	0.35	1.24	0.48	0.73	0.29	0.88	0.36	0.53	0.40	0.66	0.40	0.38	0.37	0.46	0.39
4	F	1.16	0.26	1.30	0.37	0.96	0.26	1.08	0.32	0.79	0.25	0.85	0.25	0.58	0.19	0.51	0.13
	M	1.27	0.29	1.57	0.24	1.04	0.28	1.23	0.24	0.79	0.18	0.87	0.14	0.59	0.22	0.64	0.13
5	F	1.07	0.26	1.16	0.18	0.93	0.26	1.01	0.18	0.81	0.12	0.74	0.10	0.61	0.09	0.57	0.15
	M	1.42	0.20	1.36	0.14	1.19	0.22	1.17	0.15	1.12	0.19	0.96	0.15	0.75	0.28	0.70	0.16
6	F	1.34	0.24	1.37	0.24	1.14	0.37	1.33	0.26	1.00	0.30	0.94	0.25	0.73	0.21	0.76	0.21
	M	1.66	0.22	1.71	0.22	1.28	0.30	1.38	0.22	1.06	0.16	1.05	0.16	0.82	0.20	0.85	0.13
7	F	2.29	0.36	2.33	0.56	1.91	0.36	1.96	0.41	1.50	0.30	1.58	0.35	1.08	0.20	1.21	0.25
	M	2.44	0.44	2.61	0.55	1.99	0.37	1.98	0.30	1.64	0.34	1.68	0.28	1.29	0.40	1.25	0.18
8	F	2.57	0.52	2.63	0.44	1.97	0.40	2.05	0.50	1.55	0.27	1.65	0.33	1.16	0.30	1.26	0.31
	M	2.46	0.63	2.59	0.59	2.11	0.47	2.16	0.51	1.71	0.34	1.76	0.38	1.21	0.25	1.30	0.26
9	F	2.51	0.34	2.48	0.26	1.97	0.21	2.02	0.20	1.78	0.31	1.75	0.32	1.15	0.29	1.44	0.40
	M	2.81	0.64	2.85	0.62	2.31	0.48	2.35	0.34	1.84	0.48	1.79	0.40	1.29	0.33	1.49	0.14
10	F	2.77	0.85	2.99	0.80	2.25	0.41	2.57	0.60	1.84	0.25	2.14	0.45	1.25	0.28	1.42	0.40
	M	2.94	0.66	3.07	0.62	2.29	0.40	2.43	0.39	1.88	0.31	1.96	0.28	1.40	0.29	1.55	0.28
11	F	3.17	0.74	3.41	0.61	2.55	0.49	2.65	0.63	2.24	0.42	2.30	0.44	1.58	0.32	1.55	0.39
	M	3.19	0.55	3.25	0.57	2.59	0.55	2.77	0.53	2.06	0.36	2.28	0.44	1.47	0.33	1.65	0.30
12	F	3.59	0.69	3.69	0.66	2.87	0.56	2.99	0.62	2.38	0.54	2.37	0.62	1.62	0.34	1.67	0.36
	M	3.60	0.64	3.77	0.83	3.05	0.67	3.06	0.83	2.29	0.58	2.52	0.71	1.60	0.46	1.75	0.48
13	F	3.95	0.85	3.71	0.82	3.10	0.59	3.06	0.71	2.44	0.64	2.58	0.78	1.55	0.39	1.67	0.61
	M	4.70	0.97	4.98	1.05	3.37	0.49	3.60	0.71	3.00	0.60	2.88	0.75	1.80	0.54	1.75	0.64
14	F	4.64	0.94	4.18	1.46	3.33	0.71	3.27	0.84	2.67	0.53	2.65	0.57	1.65	0.15	1.75	0.47
	M	4.83	0.97	5.37	0.92	3.90	1.03	4.09	1.03	3.24	1.03	3.21	1.05	2.05	0.55	2.03	0.62
15	F	4.59	0.41	4.29	0.43	3.33	0.30	3.29	0.49	2.70	0.19	2.56	0.53	1.66	0.31	1.78	0.27
	M	5.16	0.87	5.47	1.12	4.05	0.83	4.36	0.76	3.40	0.84	3.25	0.33	1.78	0.26	2.04	0.65
16	F	4.74	1.09	4.35	1.49	3.39	0.63	3.46	0.80	3.00	0.83	2.70	0.71	1.73	0.42	1.73	0.37
	M	5.57	1.14	6.25	1.12	4.37	0.80	4.47	0.64	3.65	0.88	3.34	0.71	1.89	0.49	2.07	0.43
17	F	4.84	0.99	4.66	0.71	4.01	0.74	4.14	0.68	2.95	0.64	2.73	0.70	1.67	0.31	1.91	0.29
	M	6.06	2.13	6.85	2.02	5.18	1.25	5.11	1.62	3.76	0.76	4.08	1.04	2.24	0.64	2.19	0.50
18	F	4.92	0.51	4.83	0.38	4.15	0.74	4.22	0.51	3.01	0.44	2.72	0.61	1.86	0.34	1.96	0.37
	M	6.83	1.92	7.10	2.35	5.89	1.08	5.57	1.95	3.95	0.34	4.10	1.59	2.39	0.45	2.25	0.46
19	F	4.93	0.72	4.89	1.03	4.16	0.43	4.24	0.93	3.09	0.50	2.91	0.56	1.78	0.30	1.96	0.22
	M	6.86	1.08	7.07	1.26	5.86	0.77	5.55	1.53	3.99	0.91	4.11	0.92	2.35	0.61	2.40	0.86

F—female, M—male, SD—standard deviation.

## Data Availability

Data are contained within the article.
